# A phase 1, first-in-human study of ^18^F-GP1 positron emission tomography for imaging acute arterial thrombosis

**DOI:** 10.1186/s13550-018-0471-8

**Published:** 2019-01-07

**Authors:** Sun Young Chae, Tae-Won Kwon, Soyoung Jin, Sun U. Kwon, Changhwan Sung, Seung Jun Oh, Sang Ju Lee, Jungsu S. Oh, Youngjin Han, Yong-Pil Cho, Narae Lee, Ji Young Kim, Norman Koglin, Mathias Berndt, Andrew W. Stephens, Dae Hyuk Moon

**Affiliations:** 1 Department of Nuclear Medicine, Asan Medical Center, University of Ulsan College of Medicine, 88, Olympic-ro 43-gil, Songpa-gu, Seoul, 05505 Republic of Korea; 2 Department of Vascular Surgery, Asan Medical Center, University of Ulsan College of Medicine, Seoul, Republic of Korea; 3 Department of Nuclear Medicine, Nowon Eulji Medical Center, Eulji University, Seoul, Republic of Korea; 4 Department of Neurology, Asan Medical Center, University of Ulsan College of Medicine, Seoul, Republic of Korea; 5 Department of Nuclear Medicine, Wonju Severance Christian Hospital, Yonsei University Wonju College of Medicine, Wonju, Republic of Korea; 6 Department of Nuclear Medicine, Guri Hospital of Hanyang University Medical Center, Hanyang University College of Medicine, Seoul, Republic of Korea; 7 Life Molecular Imaging GmbH (formerly Piramal Imaging GmbH), Berlin, Germany

**Keywords:** Arterial thrombosis, Positron emission tomography, ^18^F-GP1, Platelet activation, Glycoprotein IIb/IIIa receptor

## Abstract

**Background:**

^18^F-GP1 is a novel positron emission tomography (PET) tracer that targets glycoprotein IIb/IIIa receptors on activated platelets. The study objective was to explore the feasibility of directly imaging acute arterial thrombosis (AAT) with ^18^F-GP1 PET/computed tomography (PET/CT) and to quantitatively assess ^18^F-GP1 uptake. Safety, biodistribution, pharmacokinetics and metabolism were also evaluated.

**Methods:**

Adult patients who had signs or symptoms of AAT or had recently undergone arterial intervention or surgery within 14 days prior to ^18^F-GP1 PET/CT were eligible for inclusion. The AAT focus was demonstrated by conventional imaging within the 5 days prior to ^18^F-GP1 administration. Whole-body dynamic ^18^F-GP1 PET/CT images were acquired for up to 140 min after injection of 250 MBq of ^18^F-GP1. Venous plasma samples were analysed to determine ^18^F-GP1 clearance and metabolite formation.

**Results:**

Among the ten eligible patients assessed, underlying diseases were abdominal aortic aneurysm with endovascular repair (*n* = 6), bypass surgery and stent placement (*n* = 1), endarterectomy (*n* = 1), arterial dissection (*n* = 1) and acute cerebral infarction (*n* = 1). ^18^F-GP1 administration and PET/CT procedures were well tolerated, with no drug-related adverse events. All patients showed high initial ^18^F-GP1 uptake in the spleen, kidney and blood pool, followed by rapid clearance. Unmetabolised plasma ^18^F-GP1 levels peaked at 4 min post-injection and decreased over time until 120 min. The overall image quality was sufficient for diagnosis in all patients and AAT foci were detected in all participants. The ^18^F-GP1 uptake in AAT foci remained constant from 7 min after injection and began to separate from the blood pool after 20 min. The median standardised uptake value of AAT was 5.0 (range 2.4–7.9) at 120 min post-injection. The median ratio of standardised uptake value of AAT foci to the mean blood pool activity was 3.4 (range 2.0–6.3) at 120 min.

**Conclusions:**

^18^F-GP1 is a safe and promising novel PET tracer for imaging AAT with a favourable biodistribution and pharmacokinetic profile.

**Trial registration:**

ClinicalTrials.gov identifier: NCT02864810, Registered August 3, 2016.

**Electronic supplementary material:**

The online version of this article (10.1186/s13550-018-0471-8) contains supplementary material, which is available to authorized users.

## Background

Acute arterial thrombosis (AAT) is the proximate cause of myocardial infarction and stroke [1], collectively the leading cause of death in developed and developing countries [2, 3]. Unfortunately, prediction of adverse events from atherosclerotic plaque-related thrombus formation by comprehensive scores [4, 5] is not sufficient for individual risk assessment [6, 7]. AAT is typically triggered by the rupture of an atherosclerotic plaque and recent efforts have focused on advanced imaging methods to detect vulnerable atherosclerotic plaques that are particularly susceptible to disruption [8], thereby identifying patients who would benefit most from intensive preventive efforts [6]. However, to date, this has not translated into improved risk prediction compared with traditional approaches [9–12] and the need remains for a better understanding of imaging information and systemic risk factors for comprehensive risk assessment [7, 13, 14].

Currently, AAT is diagnosed using magnetic resonance imaging, computed tomography (CT) and angiography to visualise the obstructed blood flow, to characterise AAT after atherosclerotic plaque rupturing and to identify patients who may benefit from pharmacologic or invasive management [15, 16]. However, these approaches do not directly visualise the active pathology of AAT and identification of the culprit lesion is not always possible [17–21]. Direct imaging of AAT may increase the accuracy of detection of AAT foci associated with atherosclerotic rupture and enable clinicians to evaluate and monitor personalised pharmacologic or invasive management [22]. It may also be possible to predict adverse events resulting from plaque rupture by detecting plaque haemorrhage that has been associated with poor outcomes [12, 23]. To date, most AAT imaging has been studied in animals and is yet to translate into the clinic [22].

When an atherosclerotic plaque ruptures, platelets are rapidly recruited to the site and thrombi formed in arteries are rich in platelets [1]. ^18^F-GP1 is a new fluorine-18 labelled elarofiban derivative that specifically binds with high affinity to glycoprotein IIb/IIIa receptors on the surface of activated platelets [24]. In vitro assays have shown that ^18^F-GP1 binds to thrombi with a high clot-to-blood ratio; the binding affinity was not significantly affected by aspirin and heparin, and ^18^F-GP1 detected small AAT foci in thrombotic depositions on damaged arteries with rapid blood clearance in a monkey thrombus model [24]. More recently, ^18^F-GP1 has been successfully used in the imaging of lesions in patients with symptoms of acute deep vein thrombosis or pulmonary embolism [25].

The objectives of this study were to explore the feasibility of detecting AAT foci with ^18^F-GP1 positron emission tomography/CT (PET/CT) in patients with AAT and to quantitatively assess ^18^F-GP1 uptake. In addition, safety, biodistribution, pharmacokinetics and metabolism were assessed. To obtain the first clinical proof of concept for the detection of arterial thrombosis, patients who had undergone arterial intervention or surgery, such as endovascular abdominal aortic aneurysm repair (EVAR), were evaluated, as the endovascular stent grafts are thrombogenic and promote fibrin deposition and platelet aggregation [26–28]. These patients may, therefore, represent a clinical model of early arterial thrombosis [28]. In parallel, patients with AAT were examined with ^18^F-GP1 PET/CT to investigate AAT in clinically relevant settings.

## Methods

### Study design

A prospective, open-label, non-randomised, single-dose exploratory study was conducted to assess the safety, pharmacokinetics, biodistribution and diagnostic performance of ^18^F-GP1 PET/CT imaging in subjects with AAT or those who had undergone arterial intervention or surgery, such as EVAR. This study was conducted in parallel to a study of patients with venous thrombi that are rich in fibrin and red blood cells [25]. The study protocol was approved by the Ministry of Food and Drug Safety of Korea and the Institutional Review Board of the Asan Medical Center (Seoul, Republic of Korea). The study was conducted in accordance with the Declaration of Helsinki and institutional guidelines. All subjects provided written informed consent before participating in the study. This trial was registered at http://www.clinicaltrials.gov as NCT02864810.

The study was designed to obtain clinical proof of concept. The number of patients examined should be as low as possible for ethical reasons, but sufficiently high to avoid inconclusive results. It was, therefore, planned to enrol ten patients with AAT, plus replacements for drop-outs.

### Patients

Patients eligible for inclusion were adults (≥ 19 years) who had signs or symptoms of AAT or recent arterial intervention or surgery within 14 days prior to ^18^F-GP1 PET/CT study, thromboembolic focus demonstrated by standard imaging within 5 days before administration of ^18^F-GP1, Eastern Cooperative Oncology Group performance status of 0–2 and adequate function of major organs. Patients were excluded for the following reasons: pretreatment with glycoprotein IIb/IIIa inhibitors within 15 days before the study; chemotherapy before or within 24 h after administration of ^18^F-GP1; pregnancy; a severe, uncontrolled and/or unstable medical disease other than cancer; or any other condition or personal circumstances that might make collection of complete data difficult or impossible. Potential patients for inclusion in this study were identified at the inpatient and outpatient clinics of the Asan Medical Center (Seoul, Republic of Korea).

### Radiopharmaceutical preparation

^18^F-GP1 was synthesised by nucleophilic radiofluorination starting from the protected tosylate precursor using a non-cassette type chemistry module (TRACERlab FXFN, GE Healthcare), as previously described [24]. Each batch of ^18^F-GP1 met the criteria listed in the specification for identity, purity, radioactive concentration of ^18^F-GP1, specific activity, pH, bacterial endotoxin level and sterility. The final product was formulated as a sterile solution for intravenous injection.

### Procedures for ^18^F-GP1 PET/CT and pharmacokinetics and metabolite analysis of ^18^F-GP1

Patients were encouraged to drink a sufficient amount of water prior to ^18^F-GP1 PET/CT. No food restrictions were required. A radioactivity of 250 ± 25 MBq of ^18^F-GP1 at a total mass dose of ≤ 10 μg was administered as a slow intravenous bolus injection for up to 60 s. ^18^F-GP1 PET/CT images were acquired using a PET/CT scanner (Discovery PET/CT 690 or Discovery PET/CT 710; GE Healthcare), as previously described [25]. Serial whole-body ^18^F-GP1 PET/CT acquisition covering vertex to toe was conducted in three scanning sessions. The first time window ranged from 0 (immediately after radiotracer injection)–35 min, the second session from 60 to 80 min and the third session from 120 to 140 min. For attenuation correction of the PET scan, a low dose CT (100 kV, 20 mAs) was performed for each imaging session. Patients were asked to void their bladder before and after each imaging session.

To determine the clearance of ^18^F-GP1 and metabolite formation, venous samples were obtained at 1, 3, 10, 30, 60 and 120 min after ^18^F-GP1 injection. Radioactivity in the plasma and whole blood samples was measured using a well gamma counter (Cobra II Auto Gamma, Canberra Packard) and expressed as a percentage of the total injected ^18^F-GP1 activity per millilitre (%IA/mL). The percent fraction of the authentic non-metabolised ^18^F-GP1 in the blood was quantified by thin-layer chromatography using iTLC-SG strips (Agilent Technologies), as previously described [29]. The area under the time activity curve, peak plasma level and time taken to reach the peak level of non-metabolised ^18^F-GP1 were calculated. Urine was collected for measurement of urinary radioactivity from ^18^F-GP1 injection to 3 h post-injection.

Safety assessments included laboratory test results (Additional file 1: Table S1), vital signs, electrocardiograms and physical examinations before and 3 h after intravenous administration of ^18^F-GP1 and again at 24 h. Adverse events were recorded from the time of patient enrollment until the events were resolved, or up to a maximum of 28 days after the follow-up visit. Assessment of baseline plasma fibrinogen levels was measured based on the time for fibrin clot formation, as previously described [30], using Dade Thrombin Reagent (Siemens Healthcare Diagnostics Products) and an automated coagulation analyser Sysmex® CS-5100 system (Sysmex).

### Assessment of ^18^F-GP1 PET/CT

^18^F-GP1 PET/CT images were analysed by the consensus of two nuclear medicine physicians, who were provided with information on standard imaging, clinical and laboratory findings. The readers reviewed all images to determine whether overall image quality was adequate for interpretation. For dynamic assessment of ^18^F-GP1 biodistribution, volumes of interest (VOIs) were drawn over normal organs on dynamic fused PET/CT images. In addition, spherical VOIs with a diameter of 12 mm were centred on the maximum ^18^F-GP1 uptake of AAT lesions for the assessment of dynamic biodistribution in AAT lesions. A VOI was placed on the descending thoracic aorta to obtain blood pool information, as previously recommended [31]. Standardised uptake values (SUVs) were normalised to the injected activity and the patients’ body weight, and were defined as follows: SUV (g/mL) = (activity (Bq/mL) / injected activity (Bq)) × body weight (g). Mean standardised uptake values (SUVmean) of the VOI were obtained on each time frame to generate a time–activity profile of the ^18^F-GP1 uptake.

Analysis of AAT included ^18^F-GP1 uptake in arteries of patients who underwent arterial intervention or surgery or showed filling defects on standard imaging modalities. When lesions showed increased uptake in relation to background blood pool activity, they were regarded as positive thromboembolic foci. Maximum SUV (SUVmax) and the ratio of the SUVmax to the SUVmean of blood pool were measured of each AAT focus.

The ability of ^18^F-GP1 PET/CT for detecting AAT was assessed in terms of patient-based detection. Other lesions detected by ^18^F-GP1 PET/CT were also analysed.

### Statistical analysis

Data are expressed as median and range. A *p* value of < 0.05 was considered statistically significant. Comparison was conducted using the Wilcoxon rank-sum and signed-rank test, and Spearman’s rank correlation coefficient. All statistical tests were performed on the IBM SPSS Statistics for Windows (version 21, IBM Company).

## Results

### Patient characteristics and ^18^F-GP1 PET/CT procedures

Eleven patients were enrolled in the study and received ^18^F-GP1 between August 2016 and August 2017. One patient withdrew consent after receiving ^18^F-GP1 injection but before PET/CT acquisition; therefore, ten patients were included in the study. All were male with a median age of 72.5 years (range 44–82). The patient characteristics are listed in Table 1. The majority of patients (*n* = 6) underwent EVAR for an abdominal aortic aneurysm. The diagnosis of AAT in the remaining four patients was confirmed by CT (*n* = 3) or magnetic resonance imaging (*n* = 1). The median time interval between onset of symptoms or surgery and ^18^F-GP1 PET/CT was 6 days (range 3–7). The median administered activity was 246.1 MBq (range 240.5–251.6) and the median administered mass dose was 1.7 μg (range 0.1–3.8). Nine patients had no protocol deviation and were included in the pharmacokinetic analysis.

**Table 1:** Characteristics of the patients with acute arterial thrombosis (*n* = 10)

Characteristics	Value (range or %)
Median age, years	72.5 (44–82)
Male	10 (100)
Non-Hispanic/Latino and Asian (Korean)	10 (100)
Median body mass index, kg/m^2^	23.6 (18.9–28.4)
Underlying disease associated with arterial thrombosis	
Abdominal aortic aneurysm with EVAR	6 (60)
Bypass surgery and stent placement for peripheral atherosclerosis	1 (10)
Endarterectomy and angioplasty for peripheral atherosclerosis	1 (10)
Arterial dissection	1 (10)
Acute cerebral infarction	1 (10)
Risk factors for cardiovascular disease	
Hypertension	7 (70)
Smoking	10 (100)
Diabetes	3 (30)
Obesity	0 (0)
Hyperlipidemia	2 (20)
Medication prior to ^18^F-GP1 PET/CT	
Prior anticoagulant therapy	5 (50)
Prior antiplatelet therapy	6 (60)

*EVAR* endovascular abdominal aortic aneurysm repair, *PET* positron emission tomography, *CT* computed tomography

### Safety

^18^F-GP1 administration and PET/CT procedures were well tolerated in all patients. There were treatment-emergent adverse events of mild severity in three patients, which included symptoms or diagnostic observations due to one acute coronary syndrome, one hepatobiliary disease and one rash. The patient with acute coronary syndrome was treated with percutaneous coronary intervention 2 days before the ^18^F-GP1 injection. At the time of ^18^F-GP1 administration, most symptoms associated with acute coronary syndrome were resolved, but symptoms with mild intensity were reported. The patient with hepatobiliary disease had abnormal liver function test 10 h before the ^18^F-GP1 administration, which was not found 5 days before the enrollment to this study. The patients showed elevated liver enzymes 2 h after the ^18^F-GP1 administration. Asymptomatic skin rash was developed 6 h after ^18^F-GP1 injection in one patient. Based on chronology of the adverse events and clinical manifestations, the investigators judged that none of them were related to ^18^F-GP1, or the study procedure.

### Biodistribution and pharmacokinetics of ^18^F-GP1

**Fig. 1:**
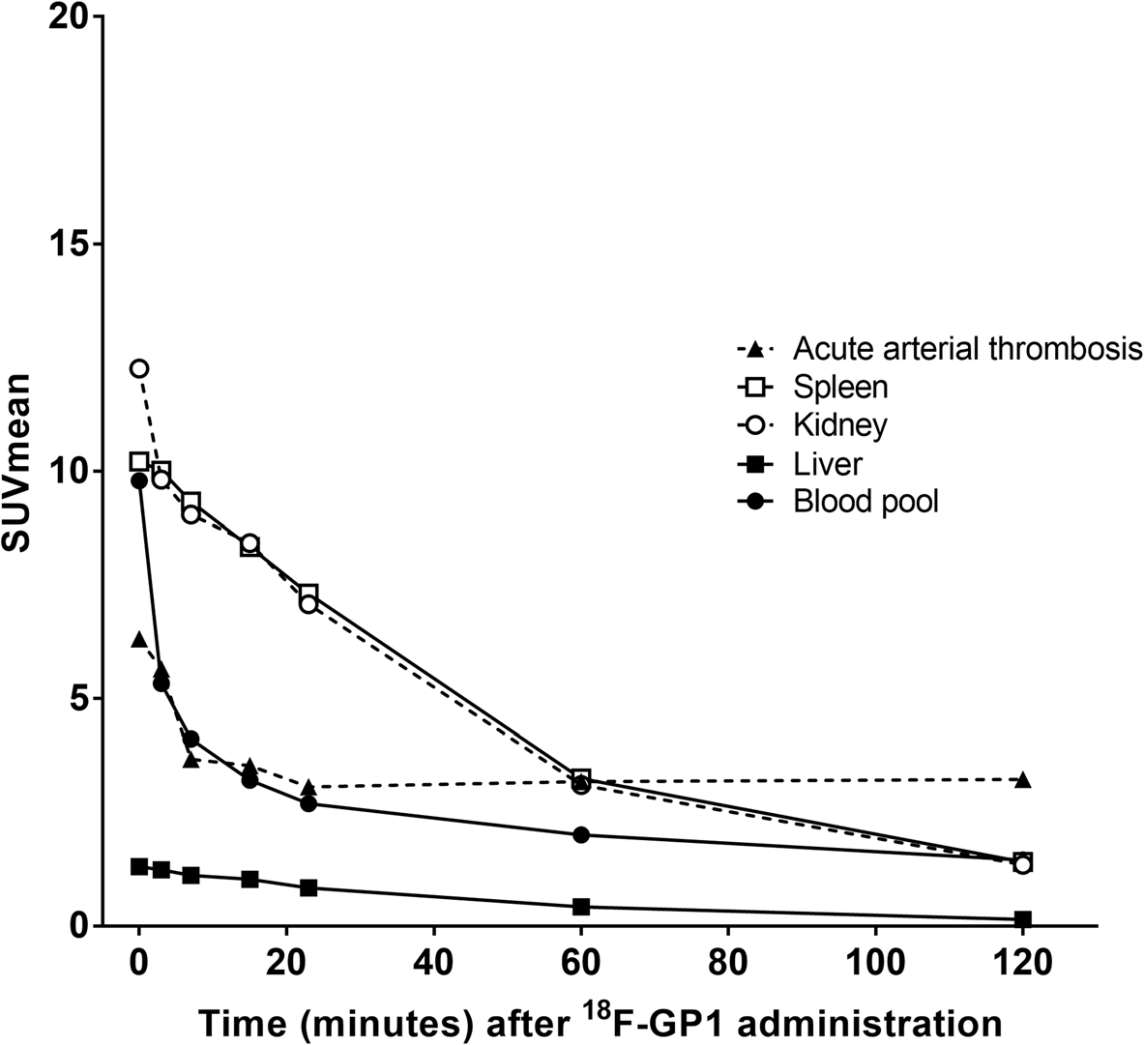
^18^F-GP1 biodistribution over time. The ^18^F-GP1 uptake in the kidney, spleen and blood gradually decreases over time. The ^18^F-GP1 uptake in AAT lesions remains constant from 7 min after injection. The median values of ten patients are shown

**Fig. 2:**
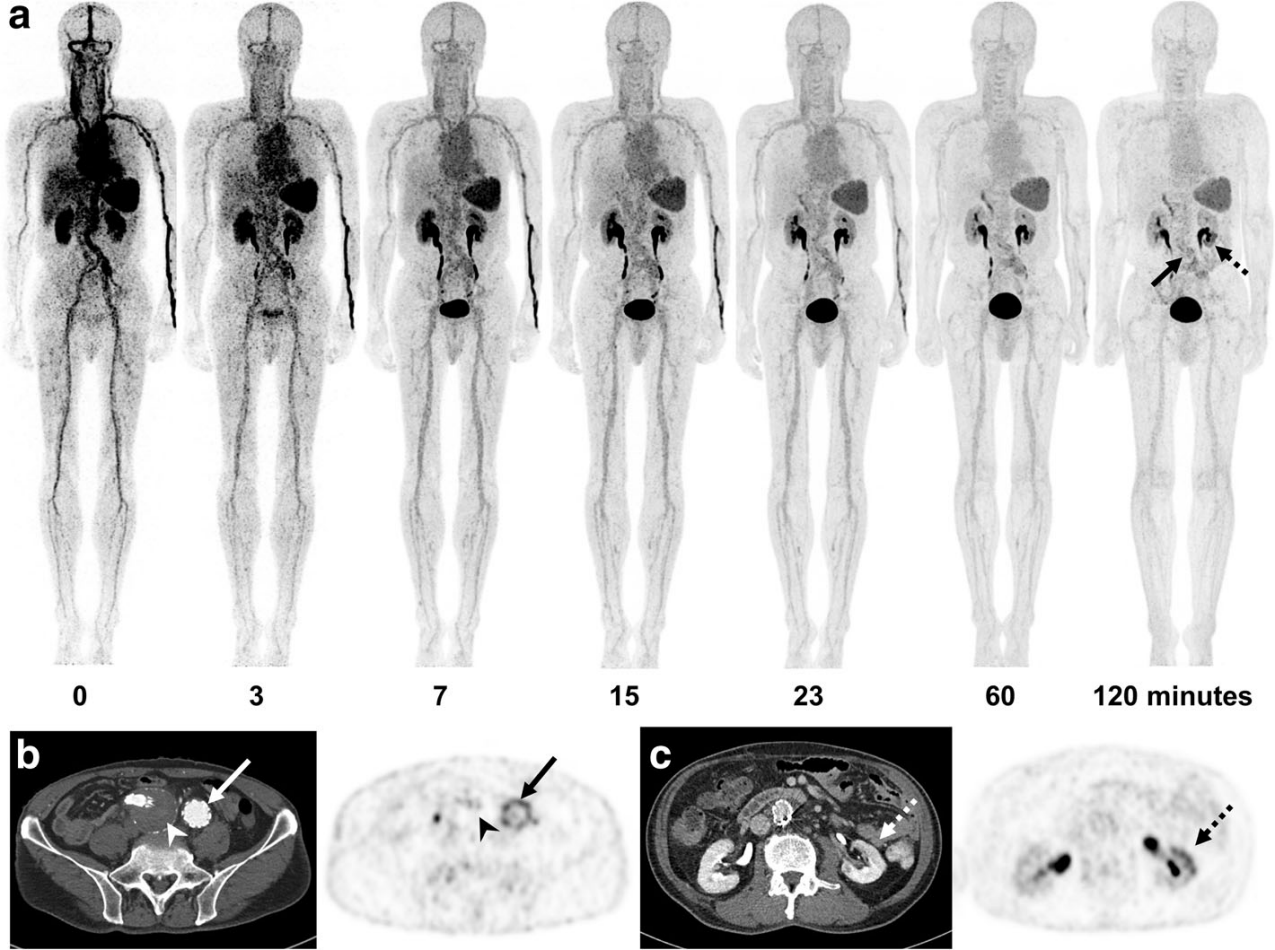
^18^F-GP1 PET/CT and CT images of a 73-year-old man who had undergone endovascular abdominal aortic aneurysm repair surgery. Anterior maximum intensity projections of ^18^F-GP1 PET/CT show positive ^18^F-GP1 accumulation in the inner surface of abdominal aortic graft (arrow) and the lower portion of the left kidney (dotted arrow), which are increasingly distinct in later images as background ^18^F-GP1 activity clears over time via urinary and hepatobiliary excretion (**a**). Transaxial CT and PET images show increased ^18^F-GP1 uptake in the inner surface of the left iliac artery graft (**b**, arrows), but no ^18^F-GP1 uptake in the chronic intraluminal thrombus in the right iliac aneurysmal sac (**b**, arrow heads). Additional positive ^18^F-GP1 uptake is observed in the non-enhancing renal ischaemic area involving the lower portion of the left kidney (**c**, dotted arrows)

All patients showed high initial ^18^F-GP1 uptake in the spleen, kidney, blood pool and liver, followed by hepatobiliary and urinary clearance (Fig. 1, Fig. 2a and Additional file 1: Table S2). The median SUVmean values in the spleen, kidney, blood pool and liver were < 1.5 at 120 min after injection. No elevated ^18^F-GP1 uptake was observed in the brain, lung, muscle or bones.

Radiolabeled metabolites were detected in only one patient with low-grade fever (38.1 °C), in whom the metabolite fraction was 12.4% at 3 min and 37.6% at 60 min after injection. The unmetabolised ^18^F-GP1 in plasma peaked at 0.0165 %IA/mL (range 0.0069–0.0253) 4 min after injection (range 2–10) and started to decrease over time until 120 min (0.0036 %IA/mL, range 0.0008–0.0049). The median peak radioactivity in whole blood was 0.0112 %IA/mL (range 0.0048–0.0182). The median area under the curve of unmetabolised ^18^F-GP1 in plasma was 1.402 %IA/mL (range 0.582–1.645). The median total radioactivity in urine was 76.2% of injected activity with a range of 35.9–81.5.

### ^18^F-GP1 uptake in AAT

The ^18^F-GP1 uptake in arterial thrombi remained constant from 7 min after injection (Figs. 1 and 2a, Additional file 1: Table S2). The median SUVmax of AAT foci was 5.3 (range 2.8–7.3) at 60 min and 5.0 (range 2.4–7.9) at 120 min (*p* = 0.29). There was no significant difference in the SUVmax of the target lesions between patients who underwent EVAR and the other four subjects who had AAT (*p* > 0.10). SUVmax was not associated with prior anticoagulation or antiplatelet treatment (*p* > 0.10). As a result of clearance from the blood pool, the ratio of SUVmax of AAT foci to blood pool increased over time to 120 min: 2.6 (range 1.5–3.9) at 60 min and 3.4 (range 2.0–6.3) at 120 min (*p* = 0.005). Elevated plasma fibrinogen was found in all ten patients (4.5 mg/mL; range 4.2–5.9). The SUVmax of AAT foci at 60 and 120 min showed no significant correlation with the fibrinogen level (*p* > 0.10).

**Fig. 3:**
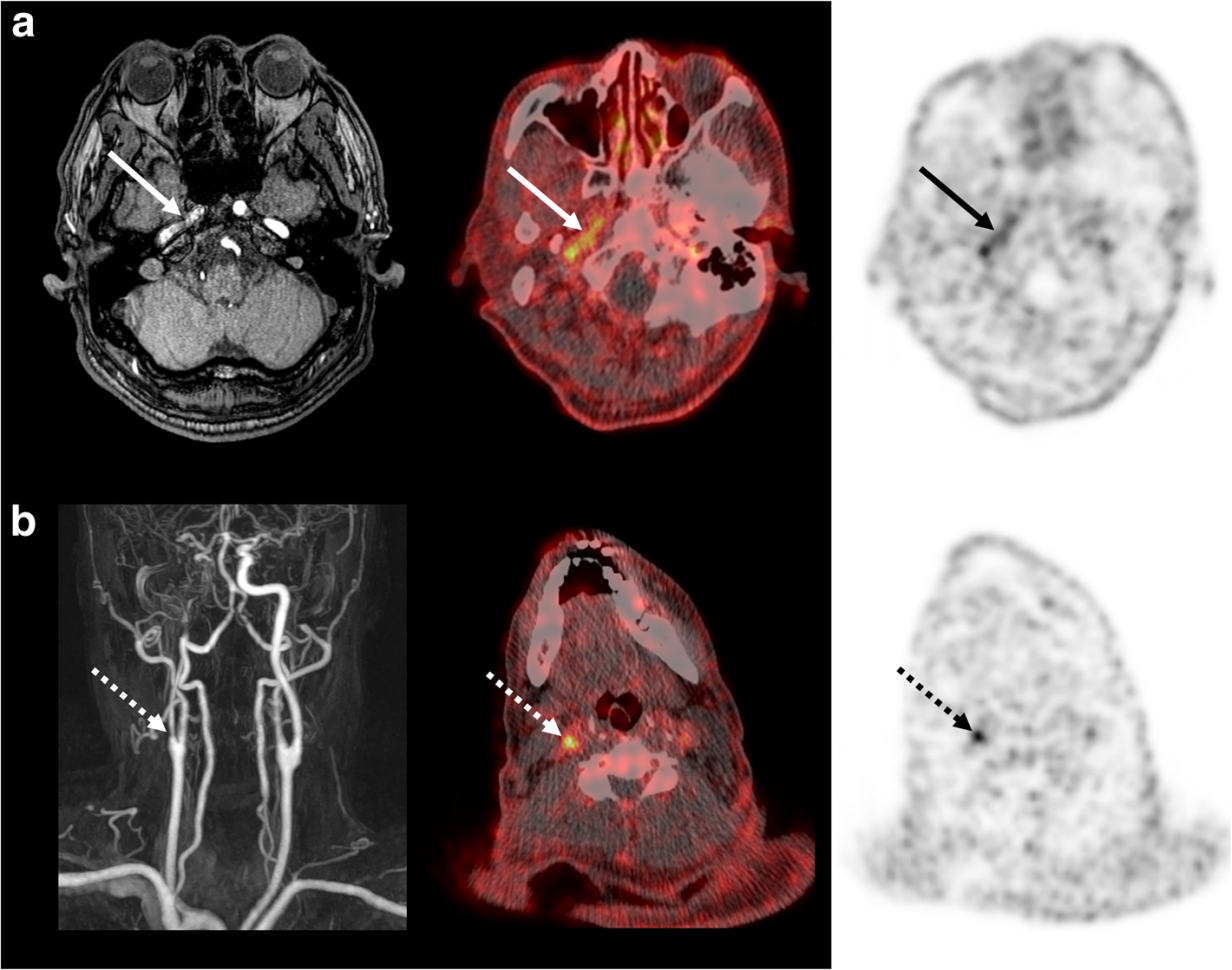
^18^F-GP1 PET/CT and magnetic resonance images of a 61-year-old man with acute cerebral infarction in the right middle cerebral artery territory and basal ganglia. Transaxial images of ^18^F-GP1 PET/CT at 60 min after injection show increased uptake in the petrous part of the right internal carotid artery (**a**, arrows) and right proximal internal carotid artery (**b**, dotted arrows). Magnetic resonance images reveal filling defects in right internal carotid artery (**a**, **b**). A smooth echogenic plaque was seen from the bilateral carotid bulbs to the proximal internal carotid arteries on transcranial Doppler (large-artery atherosclerosis subtype)

**Fig. 4:**
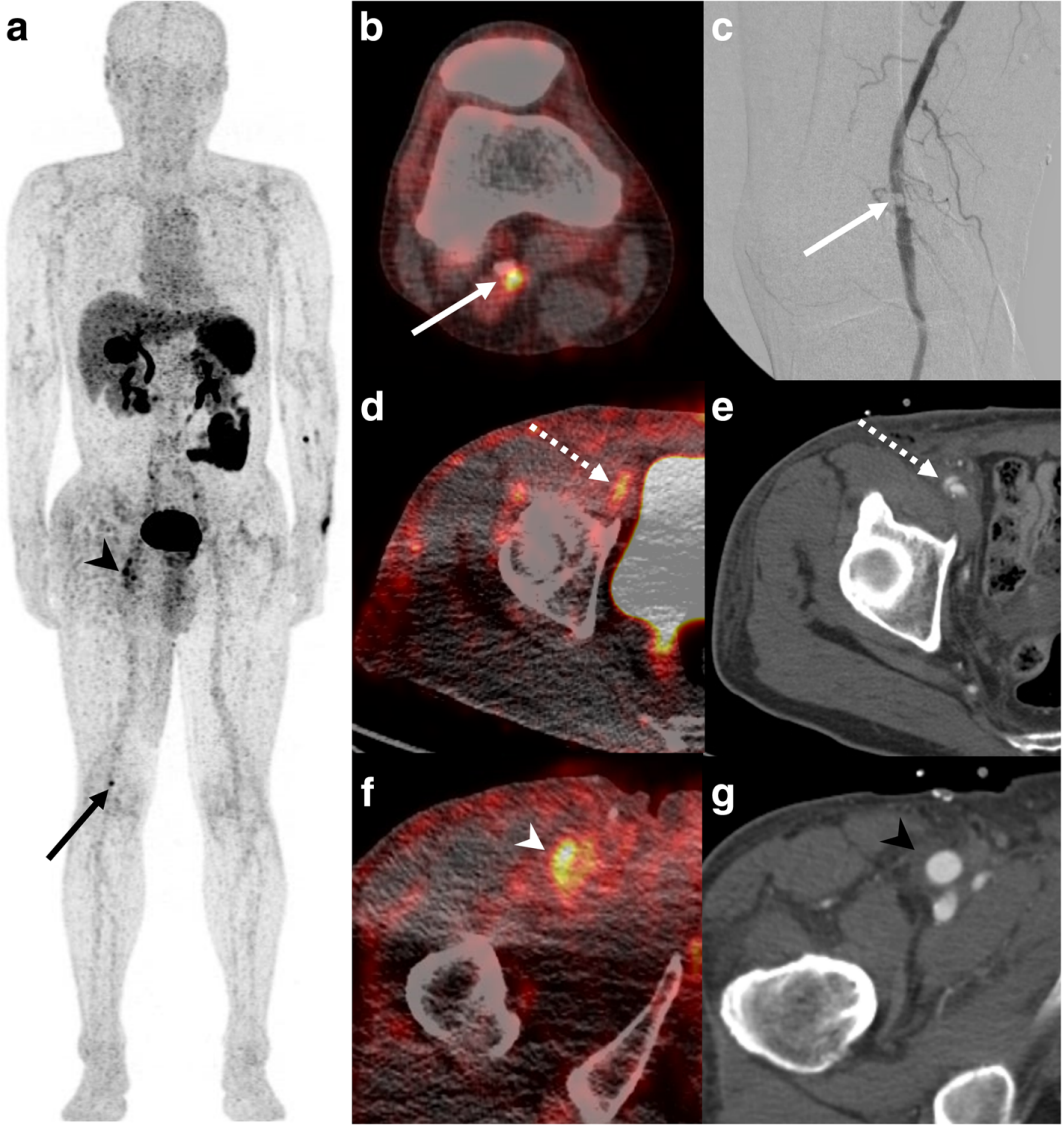
^18^F-GP1 PET/CT images of a 63-year-old man with right common femoral artery endarterectomy and right popliteal artery angioplasty. Anterior maximum intensity projection and transaxial images of ^18^F-GP1 PET/CT at 120 min after injection show a focal increased uptake in the right popliteal artery (**a**, **b**; arrows), which corresponds to the AAT lesion after angioplasty (**c**). Additional positive ^18^F-GP1 uptake is observed in the dissected right distal external iliac artery (**d**, **e**; dotted arrows) and right common femoral artery (**a**, **f**; arrow heads) where endarterectomy was performed due to occlusion 3 days before ^18^F-GP1 PET/CT (**g**, arrow head)

The image quality was adequate for interpretation in all patients. ^18^F-GP1 PET/CT distinguished between blood pool activity and AAT foci from 60 min after injection (Fig. 2a). ^18^F-GP1 PET/CT detected AAT in all ten patients, which include AAT foci in the abdominal aorta (*n* = 7, Fig. 2b), internal carotid artery (*n* = 1, Fig. 3a), superficial femoral artery (*n* = 1) and popliteal artery (*n* = 1, Fig. 4a–c). In patients with EVAR, positive ^18^F-GP1 uptake was seen on the endograft’s surface, but not in the intramural thrombus or blood trapped in the aneurysm sac. In addition, in four patients, ^18^F-GP1 PET/CT showed seven arterial lesions with increased uptake in the proximal internal carotid (Fig. 3b), middle cerebral, external iliac (Fig. 4d, e), common femoral (Fig. 4f, g) and superficial femoral arteries, as well as the femoral popliteal bypass graft (Additional file 1: Figure S1), which corresponded to ^18^F-GP1 uptake associated with atherosclerosis (*n* = 2), thromboembolic occlusion (*n* = 1), arterial dissection after surgery (*n* = 1), endarterectomy (*n* = 1), stent implementation (*n* = 1) and bypass graft (*n* = 1). Increased ^18^F-GP1 uptake was also seen in the cerebral and renal ischaemic area (Fig. 2c) and popliteal vein.

## Discussion

The data presented here represent the first human experience of ^18^F-GP1 in patients with AAT. As previously observed in patients with acute deep vein thrombosis or pulmonary embolism, ^18^F-GP1 showed a favourable biodistribution and pharmacokinetic and safety profiles. Rapid clearance from the blood pool and retention of ^18^F-GP1 uptake in delayed images allowed the diagnosis of AAT in all patients.

The symptoms and severity of AAT depends on the degree of arterial stenosis. For ethical reasons, patients who were unstable due to flow-limiting AAT were excluded and, therefore, only four patients with AAT were enrolled. The majority of patients (*n* = 6) had undergone EVAR for the treatment of an abdominal aortic aneurysm. Patients with EVAR may represent a real-life situation that involves platelet activation and aggregation during early thrombus formation, and were therefore considered suitable candidates for assessing the ability of ^18^F-GP1 to detect small thrombus formation.

The biodistribution and pharmacokinetics results of ^18^F-GP1 in patients with AAT showed clearance from major organs of the body, whereas ^18^F-GP1 uptake in AAT foci remained stable after 7 min. Thrombus-to-blood pool ratio increased over time up to 120 min due to continuous blood pool clearance. Our biodistribution and pharmacokinetic data are therefore consistent with those seen in a parallel study of patients with venous thromboembolism [25] and in animal models [24]. The high-quality imaging characteristics of ^18^F-GP1 may be due to its high specificity and affinity toward activated glycoprotein IIb/IIIa receptors on platelets [24]. The biodistribution data indicate that equilibrium was not achieved over 120 min and the specific retention of ^18^F-GP1 may be considered irreversible on the imaging timescale. Although we detected metabolites of ^18^F-GP1 in one patient, image quality and the ability of ^18^F-GP1 PET/CT to detect AAT was not affected.

AAT foci were detected in all patients using ^18^F-GP1 PET/CT up to 7 days after symptom onset regardless of prior anticoagulant or antiplatelet treatment. In patients with EVAR, we found the surface deposition of ^18^F-GP1 uptake in the endografts where thrombogenesis may persist for approximately 6 months after implantation [32]. Intraprosthetic thrombus deposition is commonly observed during follow-up for EVAR; however, it may not be associated with thromboembolic events over time [33]. Although ^18^F-GP1 uptake in the endografts may be related to mural thrombus formation, it is unlikely to be clinically relevant. No ^18^F-GP1 uptake was found in intramural thrombi or trapped blood after EVAR. It is believed that blood trapped in the aneurysmal sac during EVAR will coagulate and develop into a thrombus [34]. Therefore, the lack of ^18^F-GP1 uptake observed in the intramural thrombus or trapped blood could be because the aneurysm was concealed after EVAR.

Glycoprotein IIb/IIIa inhibitors and elevated fibrinogen can inhibit ^18^F-GP1 binding to activated platelets, which may limit the diagnostic use of ^18^F-GP1. However, currently, the use of glycoprotein IIb/IIIa inhibitors has become limited owing to high rates of bleeding complications and the development of alternative classes of antiplatelet drugs with improved safety features [35]. In addition, elevated fibrinogen did not affect ^18^F-GP1 uptake in AAT foci in this study. Therefore, it is not expected that this could compromise clinical use of ^18^F-GP1 for imaging AAT.

In this study, unexpected ^18^F-GP1 uptake was detected that may be associated with thrombotic events. One ischaemic stroke patient showed focal ^18^F-GP1 uptake in the stenotic internal carotid artery where plaque rupture may have occurred. In another patient, a focal ^18^F-GP1 uptake may be consistent with stent thrombosis. These findings suggest possible future applications for ^18^F-GP1 in imaging of atherosclerotic plaque rupture and delayed stent thrombosis. ^18^F-GP1 uptake in cerebral and renal ischaemic areas is also an area of interest and further research is required to investigate whether these findings are indicative of post-ischaemic platelet activation.

Our study has several limitations. The high detection rate should be interpreted with caution due to the small number of patients. Furthermore, the inclusion criteria and study design may not allow us to evaluate the clinical relevance of positive ^18^F-GP1 in patients. No follow-up data are available to discriminate between true or false-positive ^18^F-GP1 uptake. The efficacy and utility of this approach should be further evaluated in additional clinical trials including patients with atherosclerotic cardiovascular disease.

## Conclusions

^18^F-GP1 is a novel and promising PET tracer for imaging AAT with a favourable biodistribution, pharmacokinetic and safety profile. Despite the limited patient numbers, this first-in-human trial has demonstrated that ^18^F-GP1 was able to locate activated platelet deposition in all study subjects. Small lesions in both the carotid and peripheral vascular distributions were visualised, suggesting the possibility of diverse indications and supporting the concept that a single whole-body image could reveal multiple, perhaps unexpected, lesions. Further evaluation will be required to demonstrate the clinical utility of imaging AAT with ^18^F-GP1 PET/CT in risk stratification and the potential to select patients who would benefit from greater preventive or therapeutic measures, and monitoring personalised pharmacologic or invasive management.

## Additional file

Additional file 1: **Table S1.** Clinical laboratory parameters for safety assessment. **Table S2.** ^18^F-GP1 uptake over time. **Figure S1.** ^18^F-GP1 PET/CT images of a 76-year-old man with bypass surgery and stent placement. (PDF 499 kb)

## Abbreviations

AAT: Acute arterial thrombosis
CT: Computed tomography
EVAR: Endovascular abdominal aortic aneurysm repair
PET: Positron emission tomography
SUV: Standardised uptake value
SUVmax: Maximum standardised uptake value
SUVmean: Mean standardised uptake value
