# Supplementary material for: A phase 1, first-in-human study of 18F-GP1 positron emission tomography for imaging acute arterial thrombosis
Source: EJNMMI Res. 2019 Jan 7;9:3. doi: 10.1186/s13550-018-0471-8 (PMC6323046; doi:10.1186/s13550-018-0471-8)
Supplement: Supplementary file 1 — Table S1. Clinical laboratory parameters for safety assessment. Table S2. 18F-GP1 uptake over time. Figure S1. 18F-GP1 PET/CT images of a 76-year-old man with bypass surgery and stent placement. (PDF 499 kb) [file 13550_2018_471_MOESM1_ESM.pdf]

A phase 1, first-in-human study of ^{18}F-GP1 positron emission tomography for imaging acute arterial thrombosis

EJNMMI Research

Sun Young Chae[1]*, Tae-Won Kwon[2]*, Soyoung Jin[3]*, Sun U Kwon[4], Changhwan Sung[1], Seung Jun Oh[1], Sang Ju Lee[1], Jungsu S. Oh[1], Youngjin Han[2], Yong-Pil Cho[2], Narae Lee[5], Ji Young Kim[6], Norman Koglin[7], Mathias Berndt[7], Andrew W. Stephens[7], Dae Hyuk Moon[1]

[1]Department of Nuclear Medicine, Asan Medical Center, University of Ulsan College of Medicine, Seoul, Republic of Korea,

[2]Department of Vascular Surgery, Asan Medical Center, University of Ulsan College of Medicine, Seoul, Republic of Korea,

[3]Department of Nuclear Medicine, Nowon Eulji Medical Center, Eulji University, Seoul, Republic of Korea,

[4]Department of Neurology, Asan Medical Center, University of Ulsan College of Medicine, Seoul, Republic of Korea,

[5]Department of Nuclear Medicine, Wonju Severance Christian Hospital, Yonsei University Wonju College of Medicine, Wonju, Republic of Korea,

[6]Department of Nuclear Medicine, Guri Hospital of Hanyang University Medical Center, Hanyang University College of Medicine, Guri, Republic of Korea,

[7]Life Molecular Imaging GmbH (formerly Piramal Imaging GmbH), Berlin, Germany

Corresponding author: Dae Hyuk Moon, Department of Nuclear Medicine, Asan Medical Center, University of Ulsan College of Medicine, 88, Olympic-ro 43-gil, Songpa-gu, Seoul 05505, Republic of Korea. Tel: 82-2-3010-4592, Fax: 82-2-3010-4588, E-mail: dhmoon@amc.seoul.kr

Table S1. Clinical laboratory parameters for safety assessment

Categories	Parameters
Hematology	Erythrocytes, leukocytes, hemoglobin, hematocrit, platelets
Clotting time	Prothrombin time, reagent-independent prothrombin ratio (INR, international normalised ratio), activated partial thromboplastin time
Serum chemistry	Glutamate pyruvate transaminase, glutamate oxaloacetate transaminase, alkaline phosphatase, total bilirubin, creatinine, chloride, potassium, sodium, total protein, blood urea nitrogen, albumin
Urine analysis	pH-value, erythrocytes, leukocytes, nitrite, protein, bilirubin

Table S2. ^{18}F-GP1 uptake over time

Time,	SUVmean value[b]				
min[a]	AAT foci	Spleen	Kidney	Liver	Blood pool
0	6.3 (4.2–9.8)	10.2 (8.7–11.0)	12.3 (7.2–15.1)	1.3 (1.2–1.5)	9.8 (9.0–10.8)
3	5.7 (3.9–6.9)	10.0 (8.5–10.8)	9.8 (7.1–11.8)	1.2 (1.2–1.4)	5.3 (4.6–5.8)
7	3.7 (3.0–4.6)	9.3 (8.1–9.7)	9.0 (6.7–10.3)	1.1 (1.0–1.3)	4.1 (3.9–4.5)
15	3.5 (2.7-4.4)	8.3 (7.7–9.3)	8.4 (6.4–9.6)	1.0 (0.9–1.2)	3.2 (3.0–3.5)
23	3.1 (2.5–3.8)	7.3 (6.1–8.4)	7.1 (5.6–8.3)	0.8 (0.5–1.0)	2.7 (2.4–3.0)
60	3.2 (2.3–4.5)	3.2 (2.7–4.2)	3.1 (2.5–3.9)	0.4 (0.2–0.6)	2.0 (1.6–2.1)
120	3.2 (2.6–4.3)	1.4 (0.9–1.9)	1.3 (0.8–1.7)	0.1 (0.0–0.4)	1.5 (1.1–1.7)

[a]Time in minutes after ^{18}F-GP1 injection.

[b]Data are presented as median (interquartile range).

AAT, Acute arterial thrombosis; SUV, standardised uptake values.

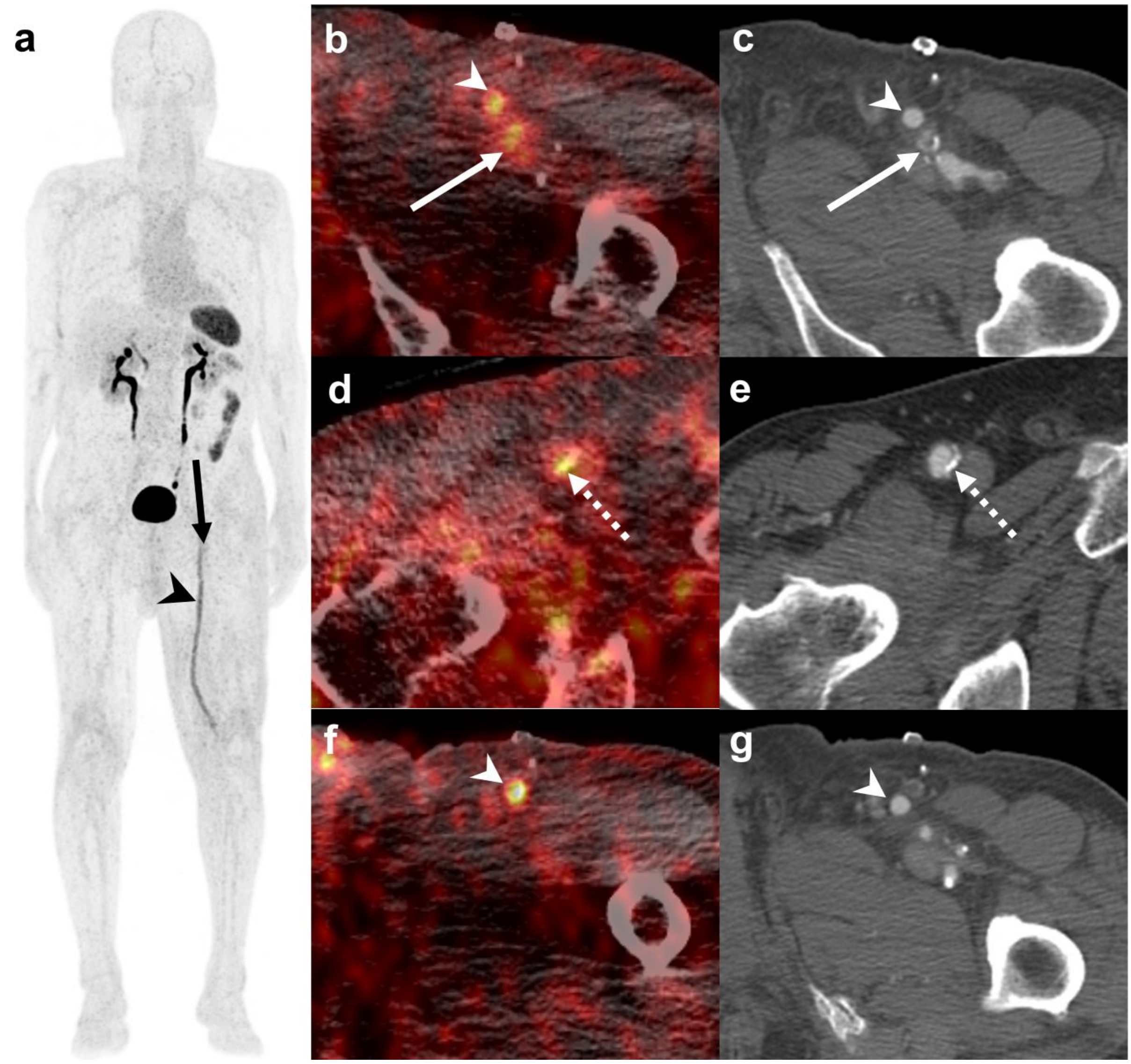

Fig. S1 ^{18}F-GP1 PET/CT images of a 76-year-old man with bypass surgery and stent placement. Anterior maximum intensity projection and transaxial images of ^{18}F-GP1 PET/CT at 120 min after injection show a focal increased uptake in the left superficial femoral artery (a and b; arrows), which corresponds to the total occlusive lesion with calcified plaque (c). Additional positive ^{18}F-GP1 uptakes are observed in the right common femoral artery with calcification (d and e; dotted arrows) and left femoral popliteal bypass graft (a, b, c, f and g; arrow heads), which was performed 3 days before ^{18}F-GP1 PET/CT.
